# Cobalt-Doped Carbon Nitride Frameworks Obtained from Calcined Aromatic Polyimines as Cathode Catalyst of Anion Exchange Membrane Fuel Cells

**DOI:** 10.3390/membranes12010074

**Published:** 2022-01-06

**Authors:** Tar-Hwa Hsieh, Sin-Nan Chen, Yen-Zen Wang, Ko-Shan Ho, Jung-Kuan Chuang, Lin-Chia Ho

**Affiliations:** 1 Department of Chemical and Materials Engineering, National Kaohsiung University of Science and Technology, 415, Chien-Kuo Road, Kaohsiung 80782, Taiwan; thh@nkust.edu.tw (T.-H.H.); n58001222@gmail.com (S.-N.C.); f108146125@nkust.edu.tw (J.-K.C.); 2 Department of Chemical and Materials Engineering, National Yu-Lin University of Science & Technology, 123, Sec. 3, University Rd., Dou-Liu City 64301, Taiwan; 3 Tri-Service General Hospital, 325 Sec. 2 Chenggong Rd., Neihu District, Taipei City 11490, Taiwan; 404010073@mail.ndmctsgh.edu.tw

**Keywords:** cobalt, aromatic polyimine, oxygen reduction reaction, anion exchanged membrane fuel cells, cathode catalyst

## Abstract

Cobalt-doped carbon nitride frameworks (CoNC) were prepared from the calcination of Co-chelated aromatic polyimines (APIM) synthesized from stepwise polymerization of p-phenylene diamine (PDA) and o-phthalaldehyde (OPAl) via Schiff base reactions in the presence of cobalt (II) chloride. The Co-chelated APIM (Co-APIM) precursor converted to CoNC after calcination in two-step heating with the second step performed at 100 °C lower than the first one. The CoNCs demonstrated that its Co, N-co-doped carbonaceous framework contained both graphene and carbon nanotube, as characterized by X-ray diffraction pattern, Raman spectra, and TEM micropictures. CoNCs also revealed a significant ORR peak in the current–voltage polarization cycle and a higher O_2_ reduction current than that of commercial Pt/C in a linear scanning voltage test in O_2_-saturated KOH_(aq)_. The calculated e-transferred number even reaches 3.94 in KOH_(aq)_ for the CoNC1000A900 cathode catalyst, which has the highest BET surface area of 393.94 m^2^ g^−1^. Single cells of anion exchange membrane fuel cells (AEMFCs) are fabricated using different CoNCs as the cathode catalysts, and CoNC1000A900 demonstrates a peak power density of 374.3 compared to the 334.7 mW cm^−2^ obtained from the single cell using Pt/C as the cathode catalyst.

## 1. Introduction

Solid-state metal element catalysts, such as Pt, need more surface area to contact with fluid reactants to perform catalysis. Consequently, it is usually prepared in nanoparticles by directly reducing Pt ions on the conducting media. Therefore, the reduction reactions for preparing Pt nanoparticles are usually carried out in the presence of conducting carbon black (CB), and this results in the so-called Pt/C products [1,2,3,4,5]. To avoid the residue of metal ions from the inorganic reducing agent, some amine-based organic compounds behave as reducing agents during Pt nanoparticle preparation through hydrothermal [6,7,8], microwave-assisted heating [9,10], or calcination [11]. However, the bulk conductivity of the Pt nanoparticle implanted matrix will be low if the implanted matrix is made of non-conducting carbon materials. We need further calcination to carbonize the non-conducting matrix to improve its conductivity by creating a more conjugative structure (more aromatic structure). Consequently, nitrogen-containing (N-containing) aromatic polymers such as polyaniline are chosen as both chelating and reducing agents for preparing Pt/C. However, high temperature calcination can result in the growth of the sizes of the implanted Pt-particles and significantly decrease the surface area as well. In other words, calcination applied to improve the conductivity of the Pt-implanted matrix is not suitable for preparing Pt-based catalysts. To avoid spending the high expense and trouble of finding suitable conducting media for precious metal catalysts like Pt, single atom catalysts (SACs), such as Fe- or Co-related compounds [12,13,14,15,16], have become promising substituents for Pt. SACs are usually prepared in the carbonaceous frameworks through calcination with metal elements covalently bonded in the carbon matrix. Metal ions, which are the active centers of the SACs, cannot chelate with neat carbon materials due to the shortage of ion-paired electrons that can coordinate with the metal ions before subjecting to calcination. Therefore, similar to the N-containing organic compounds mentioned in the preparation of Pt/C nanoparticles, we intended to polymerize Fe- or Co-ion-chelated amine-containing monomers to enclose and capture the metal ions before calcining to the SAC frameworks [17,18,19]. Eventually, Fe or Co can coordinate with four or less nitroge0n in the carbon matrix (FeNxC or CoNxC), and become the catalyzing active centers [20,21,22,23,24,25,26,27,28,29,30]. Although many macrocyclic metal organic frameworks (MOFs) containing Fe or Co centers [31,32,33,34,35,36,37,38,39] are available, they still need calcination to create conducting routes in the matrix to transport electrons; consequently, we need additional steps and effort to prepare the macrocyclic compounds. Therefore, we propose the preparation of Co-APIM with Co ions cheated with the nitrogen of the APIMs, and then pyrolyzed to become CoNC as the oxygen reduction reaction (ORR) cathode catalyst for AEMFCs.

## 2. Materials and Methods

### 2.1. Materials

PDA (Tokyo Kasei Kogyo Co., Ltd., Tokyo, Japan), OPAl (Tokyo Kasei Kogyo Co., Ltd., Tokyo, Japan), and anhydrous cobalt (II) chloride (CoCl_2_, J.T. Baker, Radnor, PA, USA) were used without further purification.

### 2.2. Preparation of CoNC Catalyst

**Scheme 1:**
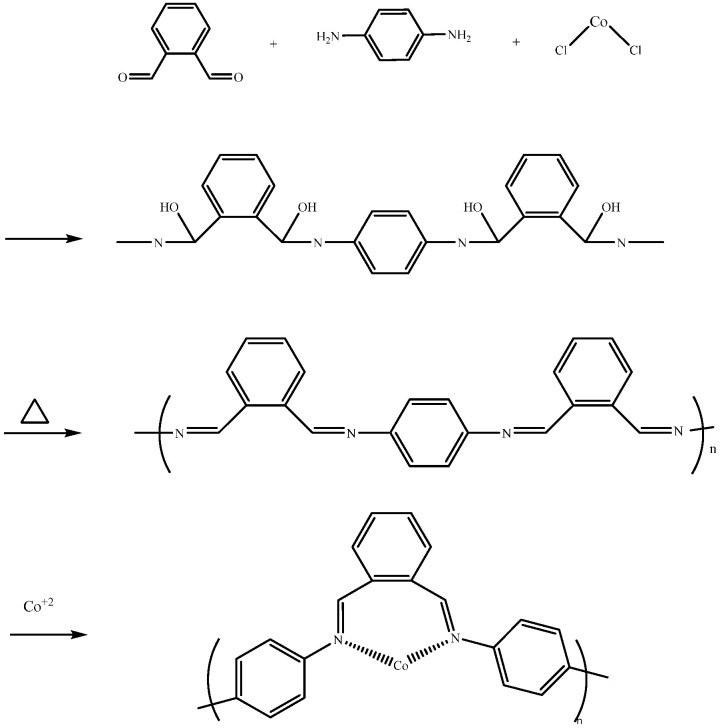
Preparations of Co-APIM.

Before mixing together to become a homogeneous alcohol solution, OPAl (1.5 g) and PDA (1 g) were dissolved in 50 and 70 mL of alcohol, respectively. A 60 mL alcohol solution of CoCl_2_ (0.65 g) was then poured into the mixture and kept stirring for 12 h at room temperature (RT). The color of the reaction mixture gradually turned into heavy orange during and after polymerization. The Co-APIM was collected by centrifugation at 3000 rpm for 10 min from the bottom precipitate of the centrifugation tube, which was dried at 80 °C for 12 h in an oven before cooling back to RT. The schematic reactions describing the synthesis of the Co-APIM are illustrated in Scheme 1.

**Scheme 2:**
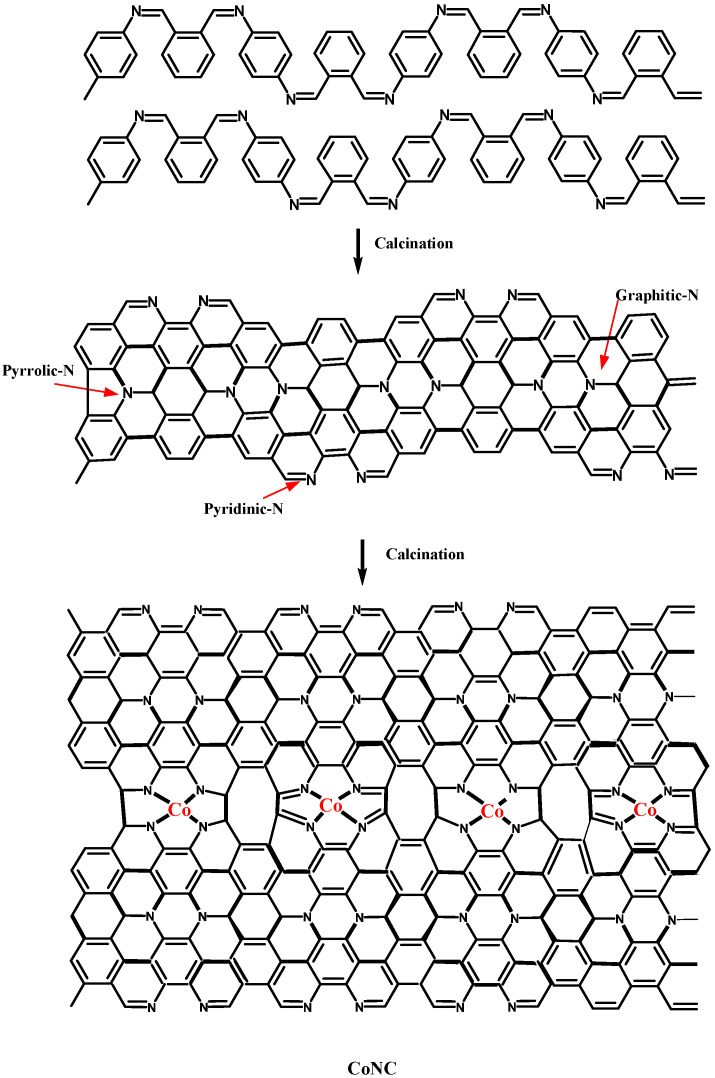
Preparations of CoNC.

Co-APIM, the temperature of the precursor of the CoNC catalyst was increased to 600 °C (700, 800, 900, and 1000 °C) at 10 °C min^−^^1^ and maintained at 600 °C (700, 800, 900, and 1000 °C) for 1 h in the ultra-pure N_2_ atmosphere, then cooled to room temperature. The impurities and magnetic compounds inside the calcined materials were removed via leaching in 2 M H_2_SO_4 (aq)_ at 60 °C for 24 h, followed by filtration. The cake was alternatively washed with de-ionized water and alcohol before drying in a vacuum oven conditioned at 80 °C for 8 h. The acid-leached products were calcined again at 500 °C (600, 700, 800, and 900 °C) in N_2_ and NH_3_ atmospheres at the same heating speed. The obtained sample was washed again in 1 M H_2_SO_4 (aq)_ at 80 °C for 3 h, followed by drying in a vacuum oven conditioned at 60 °C. The obtained sample was named as CoNC-600A500 (-700A600, -800A700, -900A800, and -1000A900). The schematic reactions describing the synthesis of the CoNCs and the formation of various types of nitrogen containing groups are illustrated in Scheme 2.

### 2.3. FTIR Spectroscopy

The characteristic functional groups belonged to PDA, OPAl, and APIM were assigned according to the FTIR spectra, which were obtained from an IFS3000 v/s FTIR spectrometer (Bruker, Ettlingen, Germany) at RT with a resolution of 4 cm^−^^1^ and 16 scanning steps.

### 2.4. Ultrviolet−Visible Spectroscopy (UV–Vis)

Samples were first dispersed in ethanol through ultrasonication before measurement. The UV−Vis spectra were obtained in a Hitachi U-2001 and DTS-1700 NIR spectrometer (Tokyo, Japan). The covering wavelength ranged from 190 to 1800 nm.

### 2.5. X-ray Photoelectron Spectroscopy (XPS)

The different binding energy spectra of N_1s_ of various CoNCs were used to characterize the percentage of nitrogen in the form of pyridine, pyrrole, graphene, Co-N., etc., after calcination with an XPS instrument assembled by Fison (VG) -Escalab 210 (Fison, Glasgow, UK) using Al Kα X-ray source at 1486.6 eV. The pressure in the chamber was kept at 10^−^^6^ Pa or less during measurement. The binding energies of the N_1s_ around 400 eV were recorded.

### 2.6. Raman Spectroscopy

The Raman spectra of all samples were obtained from a Raman spectrometer (TRIAX 320, HOBRIA, Kyoto, Japan).

### 2.7. Wide Angle X-ray Diffraction: Powder X-ray Diffraction (WXRD)

A copper target (Cu-Kα) Rigaku X-ray source (Rigaku, Tokyo, Japan), with a wavelength of 1.5402 Å, was the target for X-ray. The scanning angle (2θ) ranged from 10 to 90°, with a voltage of 40 kV and a current of 30 mA at a scanning speed of 1° min^−1^.

### 2.8. Scanning Electron Microscopy (SEM)

Using a SEM (field emission gun scanning electron microscope, AURIGAFE, Zeiss, Oberkochen, Germany), the micrometer sizes and morphologies of the CoNCs were available.

### 2.9. Transmission Electron Microscopy (TEM)

Photos of the samples were taken using an HR-AEM field-emission transmission electron microscope (HITACHI FE-2000, Hitachi, Tokyo, Japan); the samples were first dispersed in acetone, and were subsequently placed dropwise on carbonic-coated copper grids before subjecting to electron radiation.

### 2.10. Energy Dispersive X-ray Spectra (EDs)

The EDs spectra of the CoNCs were obtained from an XL-40EFG by Philips (Amsterdam, The Netherlands). The samples were coated with gold before measurement.

### 2.11. Surface Area and Pore Size Measurement (BET Method)

Nitrogen adsorption–desorption isotherms (type IV) were obtained from an Autosorb IQ gas sorption analyzer (Micromeritics-ASAP2020, Norcross, GA, USA) at 25 °C. The samples were dried in a vacuum overnight at temperatures higher than 100 °C. The surface area was calculated according to the BET equation when a linear BET plot with a positive C value was in the relative pressure range. The pore size distribution was determined with the approaches derived from the Quenched Solid Density Functional Theory (QSDFT) on a model of slit/cylinder pores. The total pore volumes were determined at P/P_0_ = 0.95.

### 2.12. Electrochemical Characterization

#### 2.12.1. Current–Potential Polarization (CV)-Linear Scan Voltammetry (LSV)

The performance of the electrocatalyst support was implemented in a three-electrode system. The round working electrode, which had an area of 1.5 cm^2^, was prepared as follows: Ag/AgCl, carbon graphite, and a Pt-strip were used as the reference, relative, and counter electrode, respectively. The electrochemical test was performed in a potentiostat/galvanostat (Autolab-PGSTAT 30 Eco Chemie, KM Utrecht, The Netherlands) in 0.1 M KOH_(aq)_ solution, and C–V curves were obtained from −0.2 to 1.0 V at a scanning rate of 50 mV s^−^^1^. The catalyst ink was prepared by mixing 2.9 mg support powder in isopropanol and stirring until uniform. Subsequently, 5% D-2020 Nafion solution (Merck, Darmstadt, Germany) was introduced into the mixture as a binder, the mixture was ultra-sonicated for 1 h, and the obtained ink was uniformly spray-coated on the carbon paper for C-V testing.

The current–potential polarization curves obtained from LSV of the various CoNCs were measured using a rotating-disk electrode (RDE: Metrohm, Tampa, FL, USA) operating at 900, 1200, 1600, 2500, and 3600 rpm in O_2_-saturated 0.1 M KOH_(aq)_, respectively. The reduction current densities of various CoNCs, which were recorded at 1600 rpm with 5 mV s^−1^ scanning speed within the measured voltage range (0.0~1.0 V), were chosen for comparison.

#### 2.12.2. MEA Preparation

**Scheme 3:**
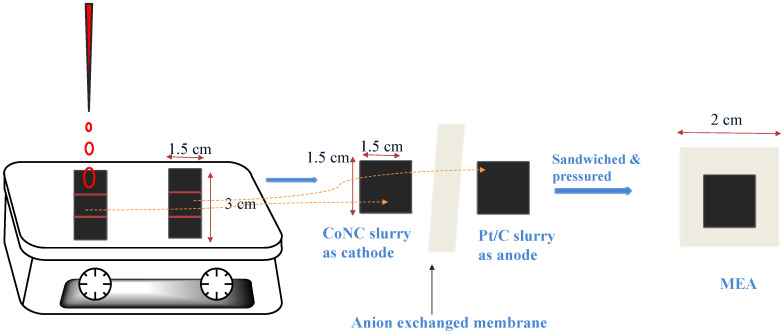
Schematic diagram for preparing MEA.

An X37-50RT sheet (50 μm), purchased from Dioxide Materials, Boca Raton, FL, USA, was used as the hydroxyl ion exchange membrane. To saturate the membranes with hydroxyl (OH^−^) ions, the X37-50RT (2 × 2 cm) membrane was submerged in 1 M KOH_(aq)_ solution for 24 h. Subsequently, the treated membranes were dipped in distilled water for 15 min and were then stored in 1 M KOH_(aq)_ solution. The catalyst inks were prepared by mixing 18 mg of CoNC powders in isopropanol and were mechanically stirred until uniform, followed by the addition of 90 mg of 5% Sustainion^®^ XB-7 alkaline ionomer ethanol solution (Dioxide Materials, Boca Raton, FL, USA), before stirring again to become uniform. Eventually, the catalyst mixture was ultra-sonicated for 1 h, followed by dropwise coating on both sides of the treated X37-50RT sheet, as the anode and cathode electrodes (2 × 2 cm), respectively, and hot-pressing at 140 °C with a pressure force of 70 kgf cm^−^^2^ for 5 min to obtain the MEA. The preparing procedures are described in Scheme 3.

#### 2.12.3. Single-Cell Performance Testing

The MEA was installed in a fuel cell test station to measure the potentials and power densities of the assembled single cell at different current densities using a single-cell testing device (model FCED-P50; Asia Pacific Fuel Cell Technologies, Ltd., Miaoli, Taiwan). The active cell area was 2 × 2 cm. The temperatures of the anode, cell, cathode, and humidifying gas were maintained at about 60 °C. The fuel-flowing rates of the anode input H_2_ and the cathode input O_2_ were set at 30 and 60 mL·min^−^^1^, respectively, based on stoichiometry.

## 3. Results and Discussion

To firmly chelate the captured cobalt ions, o-phthalaldehyde (OPAl) instead of terephthalaldehyde was chosen to condense with p-phenylene diamine (PDA) in the presence of anhydrous cobalt (II) chloride (CoCl_2_) to be Co-APIMs.

### 3.1. FTIR Spectra

**Figure 1:**
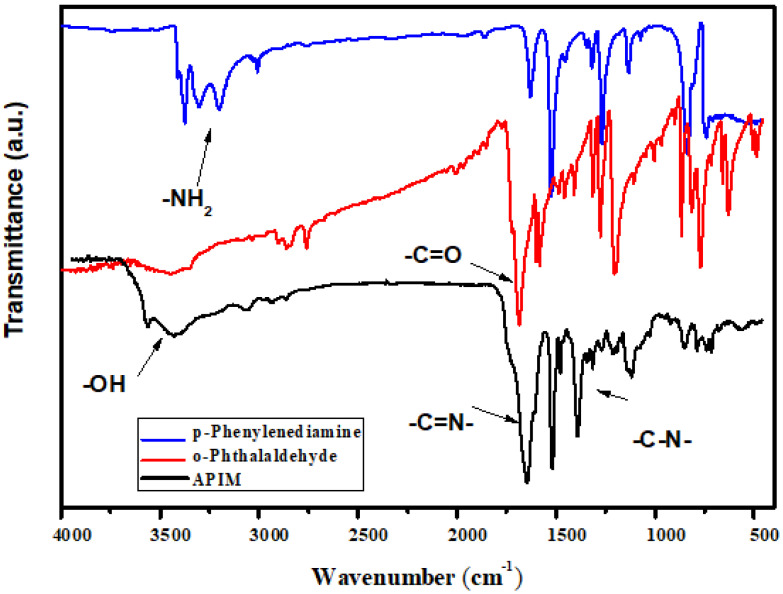
IR-spectra of PDA, OPAl, and APIM.

The IR-spectra of two types of monomers (PDA and OPAl) and APIM were illustrated in Figure 1 within the same wavenumbers ranged from 4000 to 400 cm^−1^. Two types of reactions occurred before the preparation of APIM. First is the polymerization step during which the hydrogen moves from amine to aldehyde, resulting in the connection of the two different monomers and the formation of hydroxyl groups. Followed by condensation step to remove water, the imine groups form in the backbones, which is the so-called Schiff base reaction.

In IR-spectra demonstrated in Figure 1, the primary amine of PDA contributes to the doublet peaks of 3303 and 3196 cm^−1^, which are corresponding to the asymmetric and symmetric stretches, respectively. These primary amine peaks disappear after polymerization, indicating the proceeding of Schiff reaction. The clearly seen peak around 1700 cm^−1^ is assigned as the carbonyl groups of OPAl, which was gradually replaced by the imine peak around 1620 cm^−1^ with the carrying out of condensation polymerization. The presence of hydroxyl groups around 3400 cm^−1^ means some of the reaction stops at the first step and did not go further to remove water from the backbones. The water can be removed and imine groups can form during following calcination at high temperature. The left shoulder peak next to imine one is corresponding to the unreacted carbonyl group of OPAl. It indicates aldehyde groups were excessive. Additionally, the carbon nitride single bond (–C–N–) is also seen around 1300 cm^−1^, revealing APIM was successfully obtained via Schiff base polymerization.

### 3.2. UV–Vis Spectra

**Figure 2:**
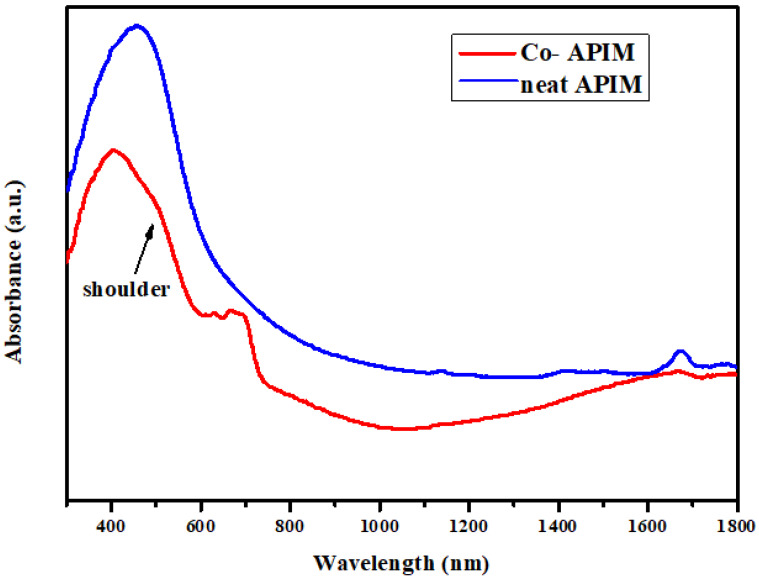
UV–Vis spectra of neat APIM and Co-APIM.

The color of APIM makes no significantly different with and without chelated Co ions. However, their UV–Vis spectra in Figure 2 demonstrated additional λ_max_ around 692 nm after complexing with Co ions and the peak that originally belongs to neat APIM (458 nm) blue-shift to 406 nm, revealing the coordination complexation between the nitrogen of APIM and Co ions did occur. The entire process is depicted in Scheme 1. The presence of the right shoulder peak of λ_max_ at 406 nm means not all APIMs are coordinated with Co ions and remain in the un-doping state (neat). Since no significant gelation phenomenon occurs after Co ion chelation, most of the coordination complexation is considered to be intramolecular (Scheme 1) instead of intermolecular.

### 3.3. XPS

The N_1S_ XPS demonstrated in Figure S1, clearly indicate the presence of cobalt nitride (Co-N) bonding, which behaves as the active catalyzing center in the cathode and other nitride compounds including pyridinic, pyrrolic, and graphitic nitrogens are all able to attract more O_2_ due to their more polar C–N bonding that can create induced dipoles on the oxygen molecules than pure C–C or C=C bonding in the matrix. In addition, the sp^3^ orbital nature of nitrogen can also create some wavy structures on the monotomous, smooth plane structures of carbon matrix to increase the surface areas. The N_1S_ XPS of CoNCs treated on various calcination conditions are illustrated in Figure S1a–d. Compositions between various nitride compounds in each CoNC catalyst are obtained by deconvoluting the N_1S_ peaks in Figure S1 and listed in Table 1. Notably, pyridinic N increase with calcination temperature and demonstrate better reduction current for the ORR, which will be discused in the electrochemical section. High percentages of pyridinic Ns mean the presence of an imperfect structure since they only exist on the edges of the frameworks, not staying inside carbon matrix like graphitic Ns. Co-N bonding can be found for all CoNCs, indicating the Co ions that coordinate with the APIM backbones already enbeded in the N-doped carbon framwork as the active centers during calcination.

### 3.4. WXAD Pattern

**Figure 3:**
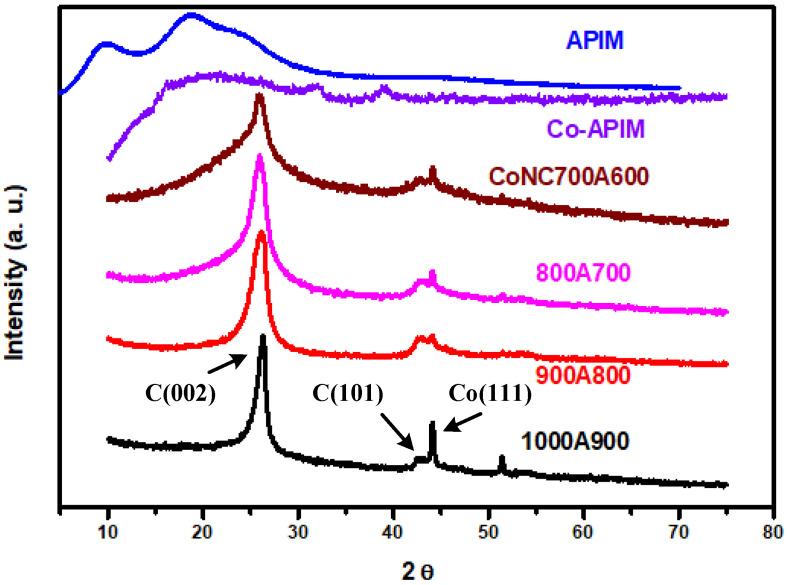
X-ray diffraction patterns of neat APIM, Co-APIM, and various CoNCs treated at different calcination conditions.

The X-ray diffraction pattern of APIM reveal it is not well-crystallized in the presence of OPAl. The ortho-substituted structure of OPAl can make the backbones of the polymers curved easily and significantly decrease the crystallinity in accordance with Figure 3. After introducing Co ions to prepare Co-APIM, the imperfect crystalline structure collapses, and the diffraction peaks seen in APIM disappear and are replaced with small peaks around 2θ = 33 and 41°, which might contribute from the residual CoCl_2_ crystals. After being subjected to calcination, beginning from 600 °C, all elements start to reorganize and develop the CoNC framework structure. Most of the carbon of the benzene rings organize into a robust conjugated honeycomb like graphene (GF) or carbon nanotube (CNT) and demonstrate characteristic diffraction peak at 2θ = 26.5° belonging to the C (002) plane, which became sharpener with temperature, as seen in Figure 2. An additional plane related to the carbon structure C (101) can be seen as well, but it is not significant. The evidence of Co-N bonding is not available in the X-ray diffraction pattern since captured Co are not able to crystallize. However, some Co elements formed and crystallized at a high temperature, contributing to the Co (111) plane in all CoNCs treated at a temperature higher than 600 °C in the N_2_ atmosphere. Actually, Co elements are not stable at high temperatures, and they can be easily bonded with other non-metal elements such as N_2_ gas at such temperatures. The presence of Co metal crystals comes from the protection of the surrounding inert carbon crystals (GF or CNT) that crystallize around Co crystal particles, preventing them from bonding with N_2_ gas (will be discussed in the TEM section).

### 3.5. Raman Spectroscopy

**Figure 4:**
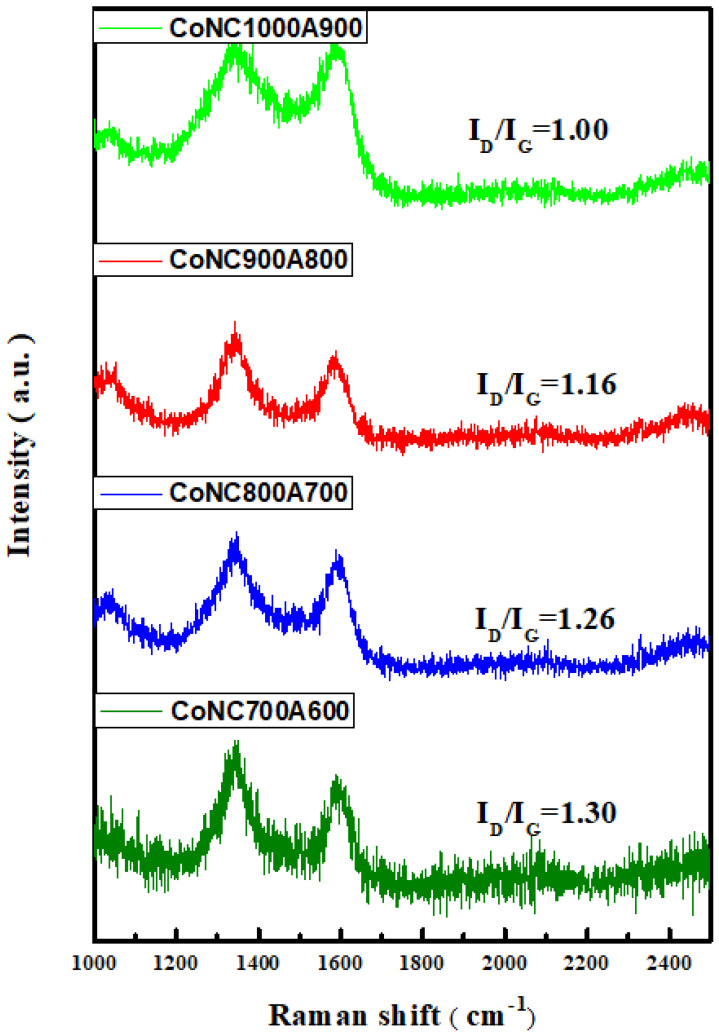
Raman spectra of CoNCs treated with various types of calcination.

During high-temperature reorganization into carbon frameworks in preparing CoNC catalysts, carbons connect with one another either in the form of sp^2^ or sp^3^ bonding. Commonly more sp^2^ carbons form with the help of high temperature from consuming sp^3^ carbons, and more conjugated aromatic structures, such as GF, are created. The characteristic peaks in Raman spectrum for sp^3^ (C–C single bond) and sp^2^ (C=C double bond) are at 1350 and 1580 cm^−1^ are named as D- and G- band, respectively. The ratio of these two peaks (I_D_/I_G_) can be used to represent the variation in the degree of order of the CoNC treated at different temperatures. The X-ray diffraction patterns illustrated in Figure 3 reveal that the C (002) peak related to sp^2^ carbon or G-band become sharpener with increasing temperatures. We also see the decreasing I_D_/I_G_-values from 1.30 to 1.00 with temperature in accordance with Figure 4, indicating more C–C is converting to C=C of GF or CNT from the calcination of APIM molecules as depicted in Scheme 2. The nitrogen of imine groups either organize into a matrix or stay at the edge as graphitic or pyridinic nitrogen as described in Scheme 2. In other words, an N-doped GF (CNT) structure is available after calcination (will be discussed in the TEM section), which provides a highly conducting matrix for the transportation of electrons from the anode via the conjugated carbon matrix. The doping nitrogen can also increase the polarity of non-polar carbon matrix in order to attract more O_2_ for the ORR. Certainly, most of the catalysis occurred around the cobalt centers that bonded with nitrogen (Co-N), as proved by XPS. The presence of the coordination element that can own six coordinated sites in the carbon matrix will leave some coordination sites empty. Consequently, when Co is perfectly embedded (doped) in the N-doped carbon framework, it will leave at least two coordination sites empty as described in Scheme 2. These two unoccupied sites can adsorb (capture) the incoming O_2_ gas molecules to induce the ORR, which will be discussed in the electrochemical section.

### 3.6. SEM Micropictures

Two-step heating was adopted to create the Co, N-doped carbonaceous framework as the cathode catalysts. The first calcination was conducted in the presence of nitrogen gas (N_2_) to create a perfectly conjugated aromatic framework, and the second step that was always performed at 100 °C lower than the first one, was to try to maintain the structure constructed in the first step. The acid-leaching carried out between the first and second calcinations can remove the crystallized Co elements or oxides on catalyst surfaces, which are not connected to the framework by primary bonding. The removal of Co nanoparticles and oxides can leave many tiny pores on the catalyst surface, as well as increasing the surface area and to expose more N- bonded Co centers to the O_2_ gas in the cathode. The magnetic attraction force that is related to the presence of Co crystals or oxides was deeply reduced after acid-leaching. In the second calcination, an additional type of gas (NH_3_) with a larger size was mixed with N_2_ in order to either create more pores, or to make up the loss of nitrogen in the first calcination.

The micrometer scale morphologies of the calcined APIM and CoNC catalysts were available in the SEM micropictures in Figure S2. In the absence of Co, the APIM molecules will crosslink and merge into large ensembles with limited surface area (smooth) after calcination at 1000 °C, referring to Figure S2a, as already described in the beginning steps of Scheme 2. The incorporation of Co brings bonding with nitrogen of the N-doped carbon matrix and gradually destroys the large ensembles into small ones with increasing temperature during the two-step calcination, in accordance with Figure S2b–f. Eventually, a coral-like morphology with a pretty high surface area and lots of tiny pores is formed for CoNC1000A900, and no large ensemble can be seen. Briefly, at higher temperature calcination, the presence of Co can significantly improve the surface area in the frameworks by creating more defect structures within the carbon nitride matrix due to its six-membered coordination, which does not match with carbon (four) or nitrogen (three). The details will be discussed in the BET section.

### 3.7. TEM Micropicture

The TEM micropictures of CoNC1000A900 were taken with different magnifications and shown in Figure S3. In smaller magnifications of Figure S2a, many ring-like morphologies are perceivable. The higher magnification micropicture of Figure S2b taken by HR-TEM is illustrated in Figure S3b, revealing that the rings seen in Figure S3a are actually made of curved CNTs that are already confirmed by X-ray diffractions and Raman spectra. In addition, there are planar structures present as well, which might belong to the GF that developed during calcination. Briefly, both GF and curved CNTs can form at high temperature calcination.

The dark spot seen in Figure S3b was further enlarged and electron diffraction was obtained to confirm the presence of the Co (111) plane seen in Figure S3c, which was also confirmed by the X-ray diffraction pattern, indicating that some element Co crystallized into particular form during calcination. The materials surrounding Co particle proved to be C (002) that originated from either GF or CNT, which protects the Co crystal from reacting with nitrogen or oxygen during crystallization.

### 3.8. EDs Spectra

**Figure 5:**
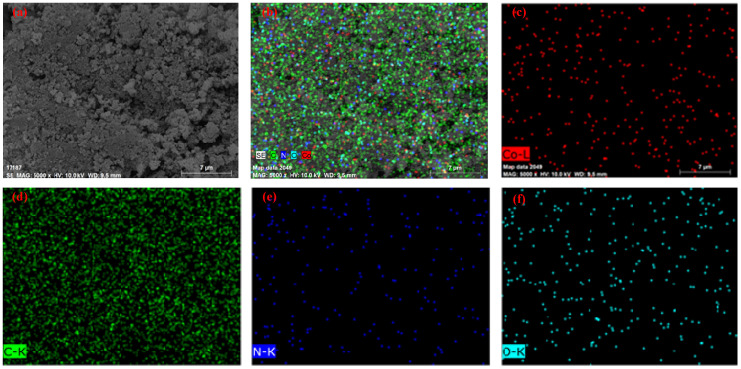
CoNC1000A900 (**a**), SEM picture (**b**), EDs spectrum and mapping pictures for (**c**) Co, (**d**) C, (**e**) N, and (**f**) O.

The SEM picture and EDs spectrum of CoNC1000A900 are illustrated in Figure 5a,b, respectively. After performing mapping for Co, C, N, and O elements, the results are posted in Figure 5c–f, respectively. According to the mapping pictures shown from Figure 5c–f, carbon dominate in the matrix and Co, N, and O are uniformly distributed in the carbon matrix. No significant Co crystal-ensemble is perceivable, indicating that almost all Co elements are removed and most of the Co is in the form of Co-Nx bonding.

### 3.9. Surface Area and Pore Size

**Figure 6:**
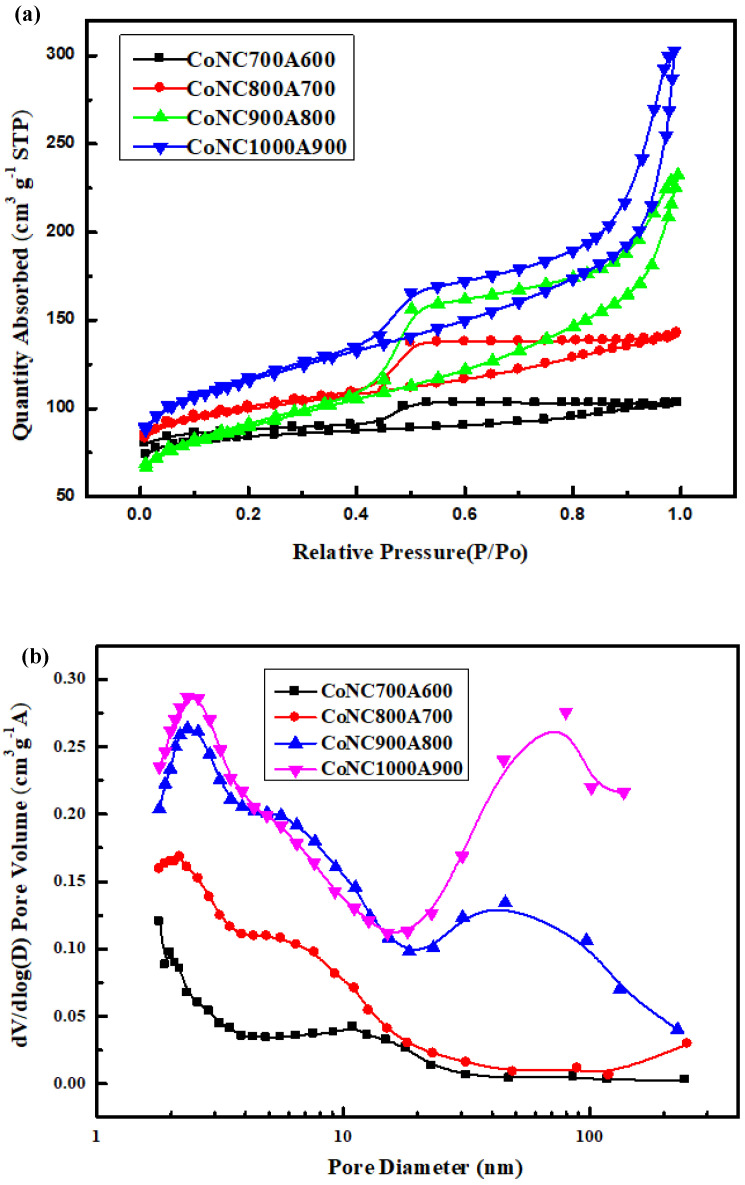
(**a**) N_2_ adsorption–desorption isotherms and (**b**) pore size distribution of CoNCs treated with different types of calcinations.

The surface area of various CoNCs were obtained by BET methods, which are demonstrated in Figure 6a and listed in Table 2. They are all type-IV isotherms resulting from the characteristic N_2_ absorption and desorption contributed from the mesopores. The pore size distribution for all CoNCs is also obtained and demonstrated in Figure 6b and listed in Table 2. The mesopore volume that is responsible for adsorbing O_2_ molecules increase with treated temperature (calcination) according to Table 2. Furthermore, CoNC1000A900 has the highest surface area of 393.94 m^2^ g^−1^, approaching 400 m^2^ g^−1^, and mainly contributed from mesopores. The SEM micropicture in Figure S2f already demonstrates a coral-like morphology with lots of pores created on the surface. The smallest surface area, which is less than 300, is found for CoNC 700A600, demonstrating large ensembles in the SEM micropicture in Figure S2c. The averaged pore sizes of CoNCs also increase with calcination temperature, referring to Table 2. With high surface area and bigger pores, CoNC 1000A900 can expose more electrocatalytic active centers to adsorb more O_2_ for carrying out the ORR at the cathode, which will be discussed in the following electrochemical sections.

### 3.10. Electrochemical Properties

#### 3.10.1. CV and LSV Curves

**Figure 7:**
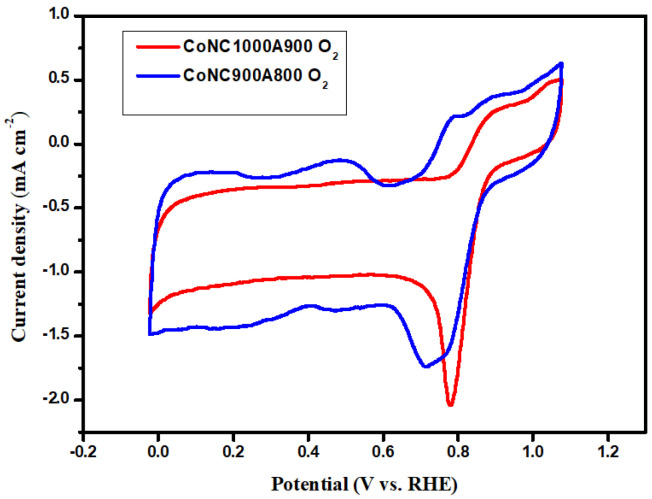
Current–potential polarization curves of CoNC900A800 and CoNC1000A900.

The current–potential polarization (CV) curve of CoNC1000A900 in Figure 7 demonstrate significantly sharp ORR peaks at 0.8 V in the O_2_-saturated KOH_(aq)_, directly indicating that it is a qualified ORR catalyst. Similar broader peak is also seen for CoNC900A800 around 0.8 V, indicating its electro-activity is still high.

**Figure 8:**
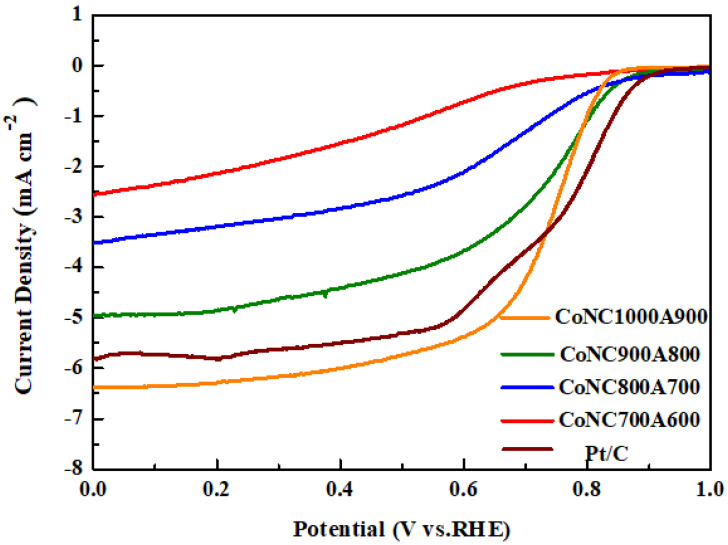
LSV curves of Pt/C and CoNCs.

The LSV curves of Pt/C and CoNCs in Figure 8 were recorded in O_2_ saturated 0.1 M KOH(aq) solution at 5 mV s^−1^ with a rotating speed of 1600 rpm. The reduction current density taken from 0 to 1 V were recorded for both Pt/C and CoNCs. The reduction current density at 0 V is called limiting current density that are also listed in Table 3. Surprisingly, the non-precious metal framework CoNC1000A900 demonstrates higher limiting current density than precious Pt/C, revealing more reduction current can supply by the cathode if CoNC1000A900 is the cathode catalyst. Other electrochemical properties related to LSV curves like onset and half-wave potentials are listed in Table 3 as well, and CoNC1000A900 still show comparable values with that of Pt/C.

**Scheme 4:**
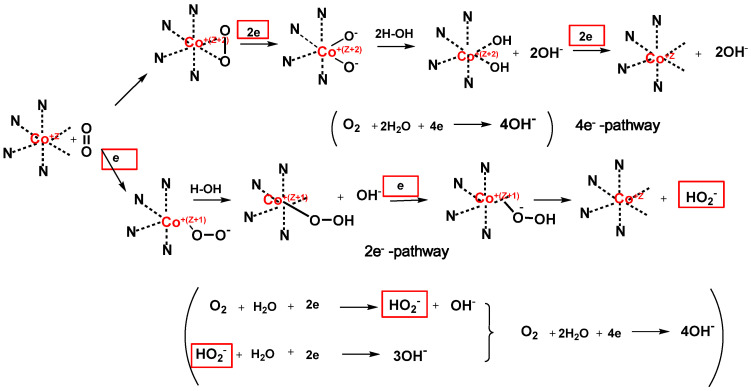
Schematic diagram of the 4e^−^ and 2e^−^ -pathways of the ORR.

Similar to the cathode of PEMFC where proton is the ions that pass through Nafion membrane, there is an unexpected side reaction occurring in the cathode of AEMFC where hydroxyl ions (OH^−^) are the ones transported from cathode to anode through the X37-50RT membrane. In other words, O_2_ does not always reduce to become OH^−^ in the cathode with water and follows the 4e^−^ -pathway described in Scheme 4, but it can also convert to an intermediate product due to incomplete reduction reaction. The intermediate is thought to be a hydrogen peroxide ion (HO_2_^−^) derived from partial ORR via the 2e^−^ -pathway. The detailed description about the formation of HO_2_^−^ is illustrated in Scheme 4. [40]

Following the 4e^−^ -pathway of ORR, O_2_ is first captured by the CoNC catalystthat is then oxidized by the captured O_2_. Upon the reduction reaction, additional electrons flow into the cathode from an anode following the external circuit and breaks the O-O bonding into two negative oxygen ions. The negative oxygen ions behave like strong base-like alkoxy ions (RO^−^), extracting hydrogen from water, which become two OH^−^ that can pass through the solid-state electrolyte membrane to the anode. The formed hydroxyl groups bonded on the surface of the CoNC catalysts can accept two additional electrons from the anode and convert to another two OH^−^ during which CoNC is turned back to the original state. Eventually, one O_2_ reacted with two H_2_O and four OH^−^ are formed following the 4e^−^ -pathway.

The unexpected reduction reaction could possibly occur if electrons are provided from the anode too slowly, or if not enough O_2_ is offered during the ORR. The double bonds of O_2_ can still be opened, but only one end will be connected to the CoNC catalyst if not enough electrons are provided. The captured O_2_ becomes peroxide ions after accepting one electron from the anode. Similarly, it will extract hydrogen from H_2_O to become a catalyst for the bonded hydrogen peroxide and the one that OH^−^ releases. After combining with another electron, it is separated from the catalyst to become an HO_2_^−^ ion. In all, there are only two electrons and one water involved in the 2e^−^ -pathway of the ORR. If HO_2_^−^ ions continue to accept one H_2_O and two additional electrons, three more OH^−^ ions will be release to follow the 4e^−^ -pathway of the ORR.

In order to calculate the numbers electrons that are transferred during the ORR for CoNC1000A900, the current density (I) was recorded at various rpms by RDE and demonstrated in Figure S4a. The reduction current demonstrated in Figure S4a can be separated into kinetic current (I_K_) and diffusion one (I_D_) in the form of Equation (1).

1/I = 1/I_K_ + 1/I_D_ (1)

By plotting K-L linear lines according to the Koutecký–Levich equation (Equation (2)), Figure S4b is constructed.

I_D_ = 0.62 × AnFD^2/3^*v*^−1/6^C√ω (2)

A—the geometric area of the disk (cm^2^);

F—Faraday’s constant (C mol^−1^);

D—the diffusion coefficient of O_2_ in the electrolyte (cm^2^ s^−1^);

*v*—the kinematic viscosity of the electrolyte (cm^2^ s^−1^);

C—the concentration of O_2_ in the electrolyte (mol cm^−3^);

ω—the angular frequency of rotation (rad s^−1^);

n—the number of electrons involved in the reduction reaction.

Therefore, the “n” numbers of electrons transferred at various potentials can be obtained from Figure S4b and can be plotted vs. potential to construct Figure S4c. The averaged *n*-value seen in Figure S4c is about 3.94, which is pretty close to the theoretical value “4” for the complete ORR.

#### 3.10.2. Single Cell Testing

**Figure 9:**
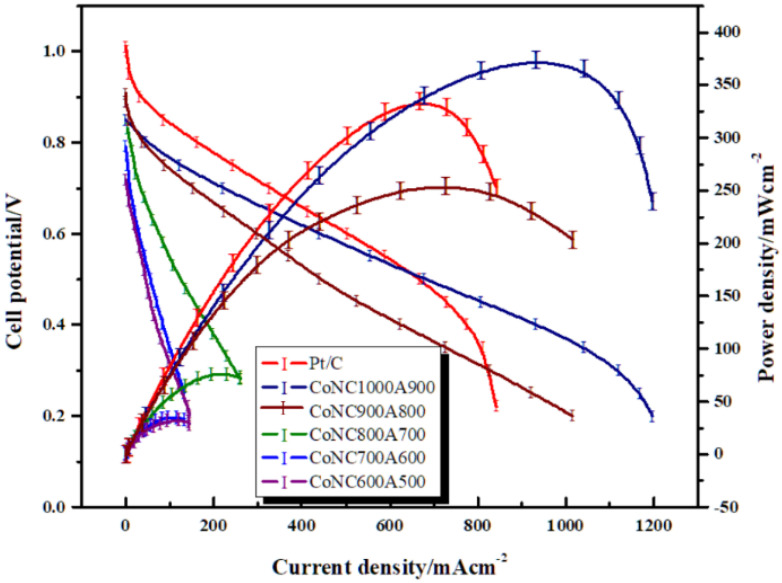
Power and current density obtained from a single cell made of CoNC cathode catalysts and treated at different calcination conditions.

The results discussed in Section 3.9 clearly reveal CoNCs can be promising cathode catalysts for the ORR. CoNCs are then mixed with Sustainion^®^ XB-7 alkaline solution to become a cathode. Combining with Pt/C as the anode catalyst and electrolyte membrane, an MEA is ready for single-cell testing in which both cell potential and power density are recorded at various current density. The results and demonstrated in Figure 9. The peak power density of Pt/C and CoNCs obtained from Figure 9 are available in Table 4. The peak power density of CoNC1000A900 cathode catalyst (374.3 mW cm^−2^) is even higher than that of commercial Pt/C catalyst (334.7 mW cm^−2^), in accordance with Figure 9 and Table 4. Even though CoNC900A800 demonstrates a less peak power density close to 250 mW cm^−2^, it is still comparable to common AEMFCs. Moreover, the power density for both CoNC1000A900 and 900A800 decay in a slower way compared with Pt/C after going over peak point according to Figure 9. Furthermore, the current density of both CoNC1000A900 and 900A800 can extend to 1000 mA cm^−2^ (1200 mA cm^−2^ for CoNC1000A900) but that of Pt/C decays very rapidly and does not go over 1000 mA cm^−2^, in accordance with Figure 9.

## 4. Conclusions

APIM was prepared via the Schiff base reaction based on the condensation between OPAl and PDA. The IR-spectra illustrate the presence of characteristic peaks of imine and other related functional groups of APIM. The chelation of Co ions in APIM induced the shifting of the main λ_max_ peaks in the UV–Vis spectrum. After calcining at various temperatures, Co-APIM became CoNC and the aromatic carbons developed into crystalline carbon matrix like GFs or CNTs by demonstrating a significant diffraction of C (002) in X-ray diffraction pattern and HR-TEM pictures. Some of the Co ions that are complexed with the imine groups of APIM crystallized during calcination to create Co (111) planes, which are confirmed by both X-ray diffraction pattern and HR-TEM pictures. For the rest of Cos that do not join the crystallization, they are covalently bonded with nitrogen in the carbon matrix, behaving as the electrocatalytic centers for the ORR in the cathode. The increasing crystallinity of CoNC based on the increasing sp^2^-carbons is proved by the Raman spectra. The surface area of CoNCs, which, concerned with the electrocatalytic efficiency, were measured by BET methods. Both surface area and average size of pores of CoNC1000A900 are found to be the highest in all CoNCs.

The CV polarization curves of both CoNC1000A900 and 900A800 demonstrated significant reduction peaks at around 0.8 V, concerned with the ORR. LSV curves of Pt/C and all CoNCs reveal that CoNC1000A900 even has higher limited reducing current than commercial Pt/C. The average number transferred during the ORR by CoNC1000A900 reached 3.94, close to theoretical value (4) for a complete ORR, indicating almost all the ORR was carried out in the catalyst following the 4e-pathway. Eventually, the power density of the single cell prepared with CoNC1000A900 as the cathode catalyst came to 374.3, compared to the 334.7 mW cm^−2^ obtained from single cell made of commercial Pt/C.

## Figures and Tables

**Table 1:** Percentages of various types of nitrogen compounds obtained from N_1s_ of XPS for different CoNCs.

	Pyridinic N	Co-N	Pyrrolic N	Graphitic N
CoNC 700A600	7.1	12.7	70	15.6
CoNC 800A700	18.6	38.2	24.2	12.1
CoNC 900A800	23.8	26.9	33.7	10.6
CoNC 1000A900	**61.6**	**25.2**	**9.1**	**4.1**

**Table 2:** BET specific areas and pore diameter of various CoNC catalysts.

CoNC	BET (m^2^ g^−1^)	Micropore (m^2^ g^−1^)	Mesopore (m^2^ g^−1^)	Total Volume (cm^3^)	Micropore Volume (cm^3^)	Mesopore Volume (cm^3^)	BJH Adsorption Average Pore Diameter (nm)
**700A600**	257.41	198.79	58.62	0.53	0.41	0.12	4.12
**800A700**	296.82	100.09	197.89	0.9	0.56	0.34	5.78
**900A800**	312.95	194.06	118.88	0.91	0.31	0.61	6.15
**1000A900**	393.94	126.31	268.89	2.03	0.65	1.39	10.34

**Table 3:** Various reduction properties concerning the ORR obtained from LSV curves for Pt/C and various CoNCs.

	Onset Potential (V)	Half-Wave Potential (V)	Limiting Current Density (mA cm^−2^)
Pt/C	**0.95**	**0.83**	**−5.29**
CoNC 700A600	0.90	0.62	−2.56
CoNC 800A700	0.90	0.70	−3.49
CoNC 900A800	0.92	0.75	−4.97
CoNC 1000A900	**0.93**	**0.77**	**−6.24**

**Table 4:** Power density of Pt/C and CoNCs.

Cathode Catalysts	Power Density (mW cm^−2^)
Pt/C	334.7
CoNC-600A500	32.2
CoNC-700A600	34.9
CoNC-800A700	76
CoNC-900A800	254.7
CoNC-1000A900	**374.3**

## Supplementary Materials

The following supporting information can be downloaded at https://www.mdpi.com/article/10.3390/membranes12010074/s1, Figure S1: N_1S_, XPS of CoNC prepared at various temperatures (a) 700A600 (b) 800A700 (c) 900A800 (d) 1000A900; Figure S2. SEM micropictures of (a) APIM-1000 (b) CoNC 600A500 (c) CoNC 700A600 (d) CoNC 800A700 (e) CoNC 900A800 (f) CoNC 1000A900; Figure S3: TEM micropictures of CoNC1000A900 taken at different magnifications; Figure S4: (a) LSV curves measured at different rpm (b) K-L lines calculated from LSV curves (c) numbers of transferred at cathode with different potentials.
