# Supplementary material for: Cobalt-Doped Carbon Nitride Frameworks Obtained from Calcined Aromatic Polyimines as Cathode Catalyst of Anion Exchange Membrane Fuel Cells
Source: Membranes (Basel). 2022 Jan 6;12(1):74. doi: 10.3390/membranes12010074 (PMC8779780; doi:10.3390/membranes12010074)
Supplement: Supplementary file 1 [file membranes-12-00074-s001.zip › membranes-1504247-supplementary.pdf]

# Supplementary Materials

# Cobalt doped carbon nitride frameworks obtained from calcined aromatic polyimines as cathode catalyst of anion exchanged membrane fuel cells

**Tar-Hwa Hsieh [1], Sin-Nan Chen [1], Yen-Zen Wang [2,*,†] , Ko-Shan Ho [1,* ,†], Rong-Kuan Jhuang [1], Lin-Chia Ho [3]**

1. Department of Chemical and Materials Engineering, National Kaohsiung University of Science and
Technology, 415, Chien-Kuo Road, Kaohsiung 80782, Taiwan; thh@nkust.edu.tw; f108146125@nkust.edu.tw
2. Department of Chemical and Materials Engineering, National Yu-Lin University of Science & Technology, 123, Sec. 3, University Rd., Dou-Liu City , Yun-Lin 64301, Taiwan
3. Tri-Service General Hospital, 325 Sec. 2 Chenggong Rd., Neihu District, Taipei City 11490, Taiwan; 404010073@mail.ndmctsgh.edu.tw

* Correspondence: wangzen@yuntech.edu.tw (Y.-Z.W.); hks@nkust.edu.tw (K.-S.H.)
† These authors equally contributed to this work.

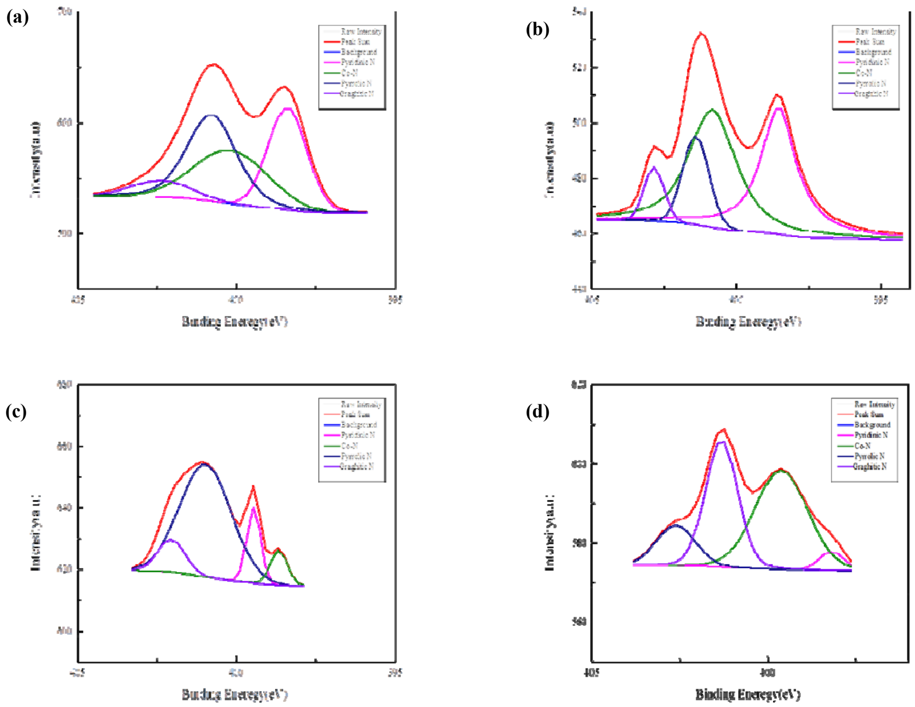


**Figure S1.** $N_{1S}$, XPS of CoNC prepared at various temperatures (a) 700A600 (b) 800A700 (c) 900A800 (d) 1000A900.

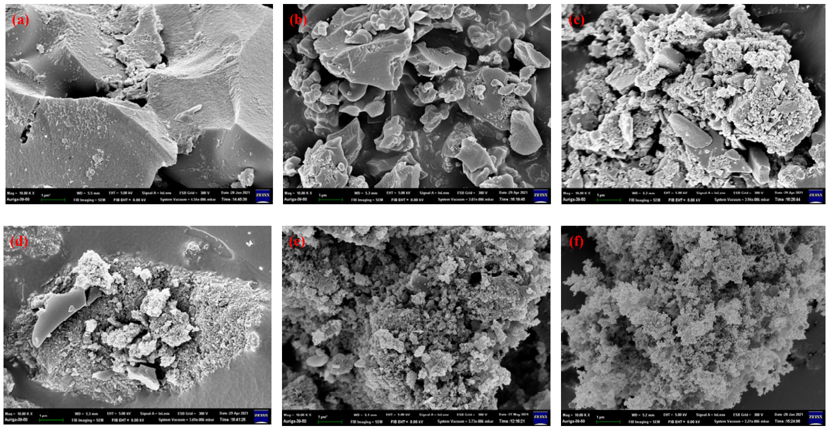


**Figure S2.** SEM micropictures of (a) APIM-1000 (b) CoNC 600A500(C) CoNC 700A600 (d) CoNC 800A700 (e) CoNC 900A800 (f) CoNC 1000A900.

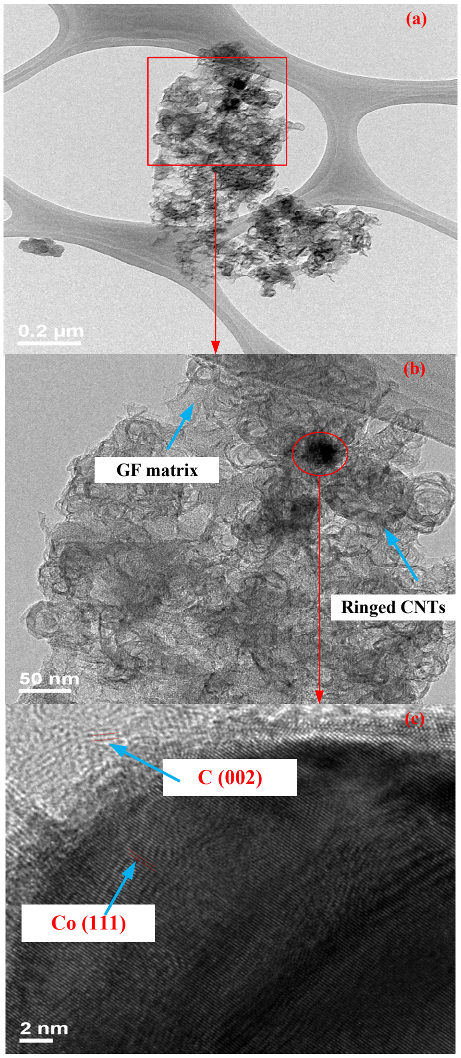


**Figure S3.** TEM micropictures of CoNC1000A900 taken at different magnifications.

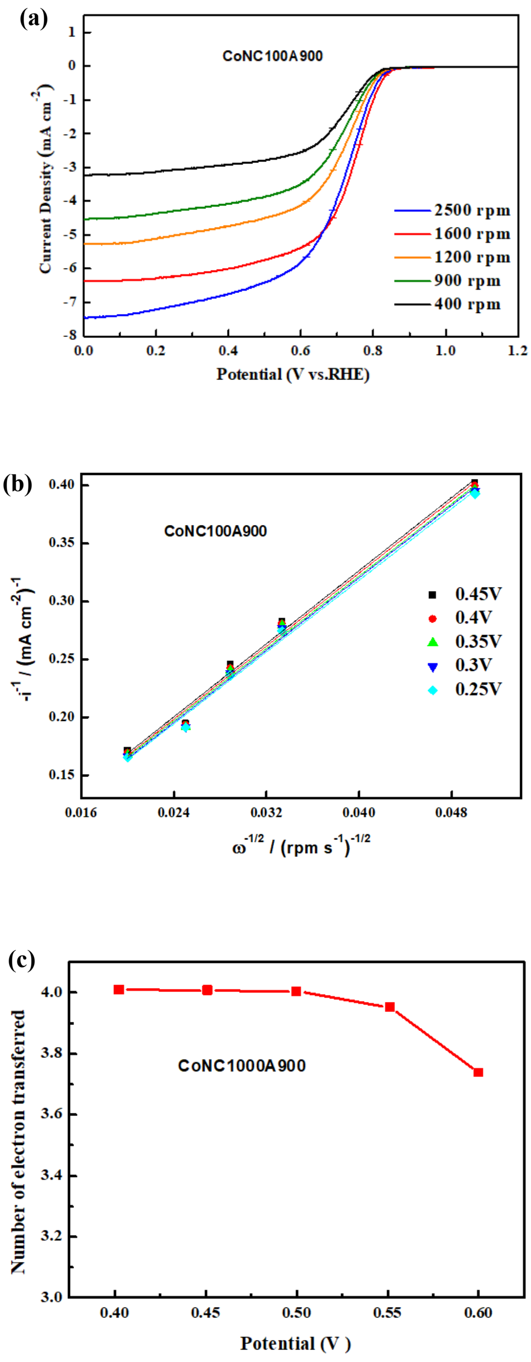


**Figure S4.** (a) LSV curves measured at different rpm (b) K-L lines calculated from LSV curves (c) numbers of transferred at cathode with different potentials.
